# *Salmonella* invasion of a cell is self-limiting due to effector-driven activation of N-WASP

**DOI:** 10.1016/j.isci.2023.106643

**Published:** 2023-04-12

**Authors:** Anthony Davidson, Peter J. Hume, Nicholas P. Greene, Vassilis Koronakis

**Affiliations:** 1 Department of Pathology, University of Cambridge, Cambridge, UK

**Keywords:** Molecular biology, Microbiology, Cell biology

## Abstract

*Salmonella* Typhimurium drives uptake into non-phagocytic host cells by injecting effector proteins that reorganize the actin cytoskeleton. The host actin regulator N-WASP has been implicated in bacterial entry, but its precise role is not clear. We demonstrate that Cdc42-dependent N-WASP activation, instigated by the Cdc42-activating effector SopE2, strongly impedes *Salmonella* uptake into host cells. This inhibitory pathway is predominant later in invasion, with the ubiquitin ligase activity of the effector SopA specifically interfering with negative Cdc42-N-WASP signaling at early stages. The cell therefore transitions from being susceptible to invasion, into a state almost completely recalcitrant to bacterial uptake, providing a mechanism to limit the number of internalized *Salmonella*. Our work raises the possibility that Cdc42-N-WASP, known to be activated by numerous bacterial and viral species during infection and commonly assumed to promote pathogen uptake, is used to limit the entry of multiple pathogens.

## Highlights

• The *Salmonella* effector SopE2 drives N-WASP activation via Cdc42
• N-WASP-driven actin assembly inhibits *Salmonella* uptake
• SopA interferes with N-WASP signaling to permit initial *Salmonella* entry

## Graphical abstract

**Figure:**
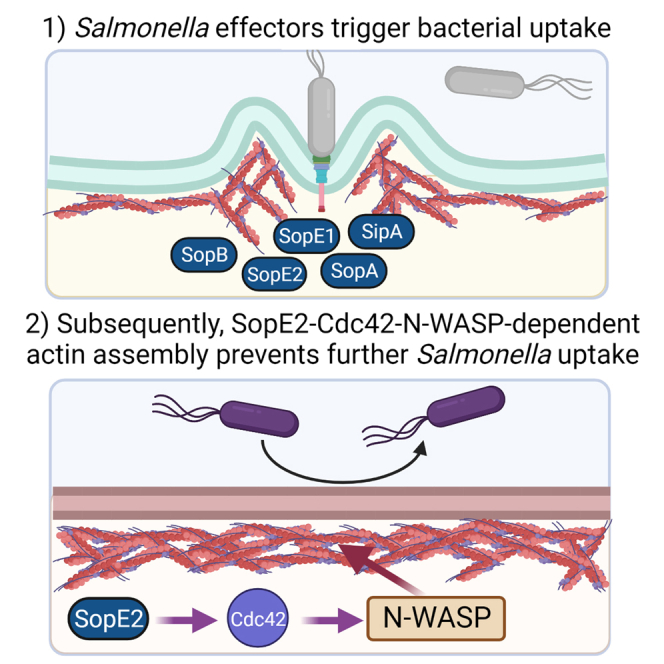

## Introduction

*Salmonella enterica* is a facultative intracellular bacterial pathogen that causes a range of diseases in both humans and animals.^1^ *S. enterica* serovar Typhimurium (*S*. Tm) typically causes acute inflammatory gastroenteritis but can also induce a more serious systemic disease.^2^ Once ingested through e.g. contaminated food, *S.* Tm takes advantage of the host’s inflammatory response to gain a competitive advantage over the resident microbiota in the gut lumen.^3^^,^^4^ This inflammatory response is driven by the invasion of non-phagocytic intestinal epithelial cells by a fraction of the infecting pathogen population.^5^^,^^6^ *S.* Tm has a multitude of likely redundant mechanisms to stimulate actin-dependent uptake into cells.^7^

The best-characterized mode of *S.* Tm entry into epithelial cells uses a type 3 secretion system to inject a myriad of effector proteins that manipulate and mimic host proteins involved in the regulation of the actin cytoskeleton.^8^ For example, the delivered effectors SipA and SipC directly bind actin, cooperatively nucleate actin polymerization and bundle actin filaments.^9^ In contrast, SopE1 and SopB manipulate the cytoskeleton indirectly^10^—SopE1 is a guanine nucleotide exchange factor (GEF), activating host GTPases such as Rac1,^11^ while SopB has lipid phosphatase/phosphotransferase activity^12^^,^^13^ that results in the recruitment and activation of the GTPase Arf1.^14^ Rac1 and Arf1 cooperate to activate the WAVE regulatory complex (WRC),^14^^,^^15^ one of the cell’s major nucleation promoting factors (NPFs), that stimulate the activity of the branched-chain actin nucleator the Arp2/3 complex. The resulting generation of distinct actin-rich membrane protrusions^16^ encapsulate and internalize the bacteria in a manner similar to macropinocytosis.^17^ A second NPF, WASH, has also been shown to play a role in the actin rearrangements underlying cell invasion, though the precise mechanism by which *S.* Tm manipulates this pathway remains uncertain.^18^

Perhaps the best characterized cellular NPF is N-WASP, which is activated by the GTPase Cdc42.^19^ N-WASP is recruited to *S.* Tm entry foci,^20^ and Cdc42 is activated during invasion by effectors including SopE1 and its homolog SopE2.^21^^,^^22^ It is therefore widely assumed in the literature that, like the other NPFs described above, N-WASP promotes pathogen uptake into epithelial cells. However, there is little evidence of this and in N-WASP knockout cells, *S.* Tm uptake is moderately enhanced, suggesting N-WASP may be inhibitory to pathogen entry.^18^ The notion that *S.* Tm might recruit a host factor that would restrict its own uptake into cells is intriguing, therefore we sought to investigate exactly what role N-WASP plays in *S.* Tm infection.

## Results

### N-WASP inhibits *Salmonella* uptake

**Figure 1:**
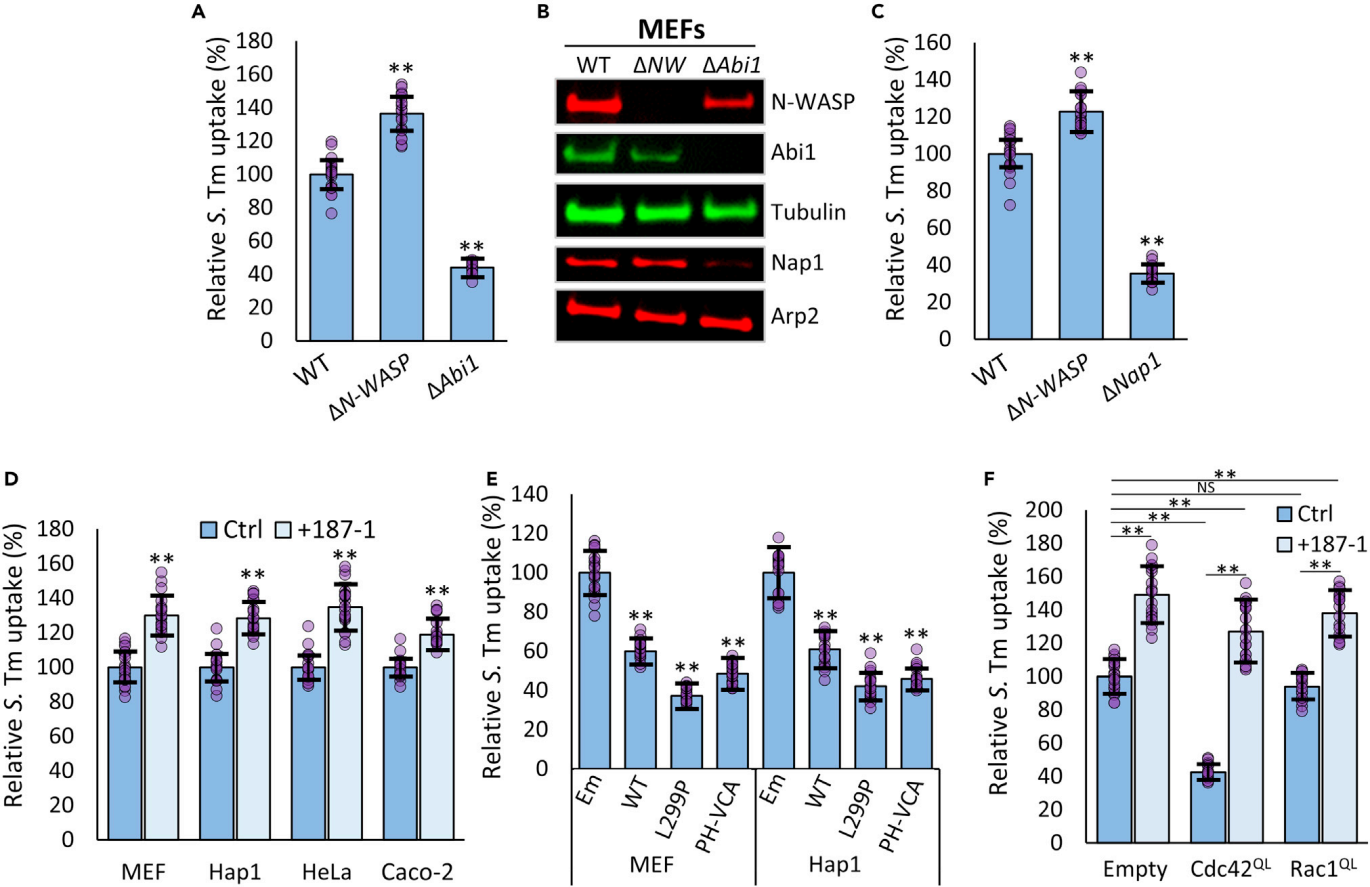
N-WASP inhibits *Salmonella* uptake (A) Uptake of *S.* Tm into WT, Δ*N-WASP,* and Δ*Abi1* MEFs. All values relative to uptake into WT MEFs. (B) Immunoblot of WT, Δ*N-WASP* (ΔNW) and Δ*Abi1* MEF cell lysates depicting relative levels of N-WASP, Abi1, Nap1, Arp2, and loading control tubulin. (C) Uptake of *S.* Tm into WT, Δ*N-WASP* and Δ*Nap1* Hap1 cells. All values relative to uptake into control WT Hap1 cells. (D) Uptake of *S.* Tm into WT MEF, WT Hap1, WT HeLa and WT Caco-2 cells that were pretreated with PBS (Ctrl) or 10 μM of the N-WASP inhibitor 187-1 (+187-1). All values relative to respective control cells. (E) Uptake of *S.* Tm into WT MEFs or Hap1 cells ectopically expressing an empty control vector (Em), N-WASP, N-WASP^L299P^, or PH-VCA. All values relative to WT Hap1 or MEFs transfected with a control empty vector. (F) Uptake of *S.* Tm into WT MEFs ectopically expressing a control empty vector (Empty), Cdc42^QL^ or Rac1^QL^. Cells were pretreated with PBS (Ctrl) or 10 μM 187-1 (+187-1). All values relative to MEFs transfected with a control empty vector. All *S.* Tm uptake was measured 30 min after initial infection. Values are the means of three independent replicates (each replicate is comprised of 6 fields of view, n = 18. Each field of view is approximately 50-100 cells, see method details). Error bars indicate standard deviation. NS – no significant difference, ∗∗ - p < 0.01 (ANOVA followed by post hoc Dunnett’s comparison).

To establish the contribution of N-WASP to *S.* Tm uptake, invasion assays were performed in Mouse Embryonic Fibroblasts (MEFs) lacking N-WASP *(*Δ*N-WASP*) or control cells lacking the WRC component Abi1 (Δ*Abi1*). Whilst the loss of Abi1 results in a 55% decrease in *S.* Tm uptake, as reported previously,^18^ the loss of N-WASP promotes invasion by over 35% (Figure 1A). Immunoblotting confirmed that relative to control tubulin expression, N-WASP knockout did not affect the expression of the WRC components Abi1 or Nap1, or the Arp2/3 complex component Arp2. Likewise Abi1 knockout did not affect N-WASP or Arp2 expression, though did reduce the expression of Nap1, as has been previously demonstrated^23^ (Figure 1B). A similar increase in *S.* Tm entry was observed in the near haploid, human cell line Hap1 lacking N-WASP (Δ*N-WASP*) (Figures 1C and S1A), and also in WT MEF and Hap1 cells treated with the N-WASP inhibitor 187-1^24^ (Figures 1D and S1B). We also utilized two popular cell models for the study of enteric infectious disease, the widely available cervical epithelial cell line HeLa and the enterocyte-derived Caco-2, which exhibits features such as tight junction-mediated barrier function, apical brush border formation and elevated expression of intestinal enzymes, receptors and transporters.^25^ In both cases, treatment with 187-1 led to a comparable increase in *S.* Tm entry to that seen for Hap1 and MEF cells (Figure 1D). As expected, the addition of 187-1 to Δ*N-WASP* MEF or Hap1 cells had no additional effect, however 187-1 treatment of both Δ*Abi1* MEFs and Δ*Nap1* Hap1 cells led to a significant increase in *S.* Tm cell entry (Figure S1C). Therefore, it appears that N-WASP inhibition actually enhances both WRC-dependent and -independent uptake of *S.* Tm.

Consistent with this inhibitory role, ectopic expression of wild-type (WT) N-WASP in either WT MEF or Hap1 cells (Figure S1D) impeded *S.* Tm uptake by approximately 40%, and the expression of a constitutively active N-WASP mutant (N-WASP^L299P^)^26^ caused an even greater defect (Figure 1E). Expression of the Arp2/3 complex-binding VCA domain of N-WASP (VCA) caused a 20% inhibition, but when fused to a phosphoinositide-binding pleckstrin homology (PH) domain (that localises the VCA to the plasma membrane—PH-VCA, see method details) uptake was more severely impaired (Figure 1E). Likewise, expression of a constitutively active form of the N-WASP activator Cdc42 (Cdc42^QL^) impeded *S.* Tm uptake by approximately 70%, an effect completely reversed in the presence of the N-WASP inhibitor 187-1 (Figure 1F). Conversely, the expression of control WRC-activating Rac1^QL^ had no significant effect on *S.* Tm uptake. Collectively these results demonstrate that N-WASP, activated by Cdc42, is inhibitory for *S.* Tm entry into mammalian cells.

### N-WASP-dependent inhibition of *Salmonella* uptake is driven by SopE2

**Figure 2:**
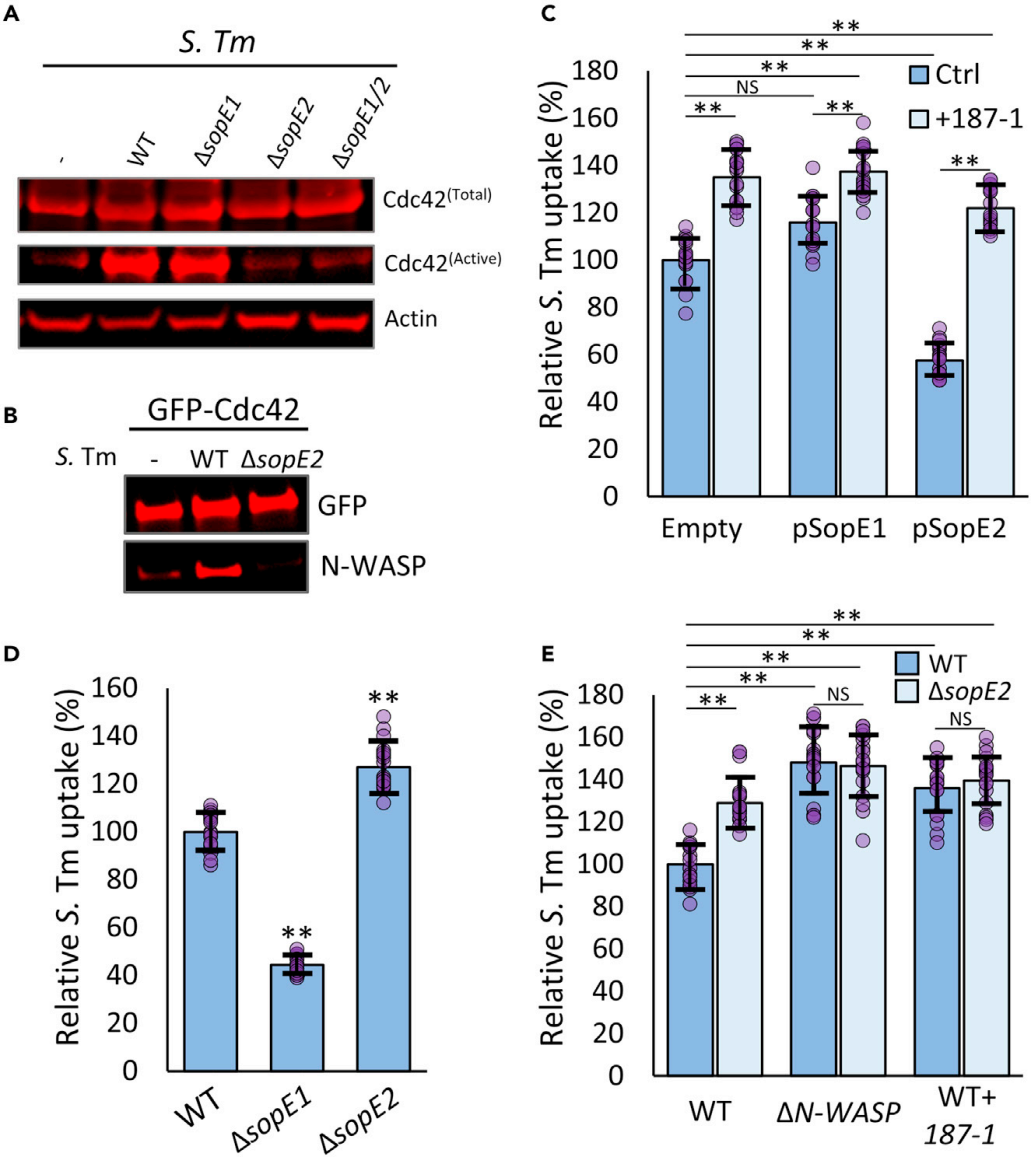
N-WASP-dependent inhibition of *Salmonella* uptake is driven by SopE2 (A) Immunoblot of GST-PBD-isolated “active” Cdc42, total Cdc42 from cell lysates and actin as a loading control. Lysates were obtained from WT MEFs that were uninfected (−) or infected for 30 min with WT, Δ*sopE1*, Δ*sopE2* or Δ*sopE1/E2 S.* Tm, as indicated. (B) Immunoblot of N-WASP interaction with GFP-Cdc42. GFP-Cdc42 was ectopically expressed in WT MEFs 48 h prior to isolation by GFP-Trap. Cells were uninfected or infected for 30 min with WT or Δ*sopE2 S.* Tm. (C) Uptake of WT *S.* Tm into WT MEFs ectopically expressing a control empty vector (Empty), SopE1 (pSopE1), or SopE2 (pSopE2). Cells were pretreated with PBS (ctrl) or 10 μM 187-1 (+187-1). All values relative to WT MEFs transfected with a control empty vector. (D) Uptake of WT, Δ*sopE1*, and Δ*sopE2 S.* Tm into WT MEFs. All values relative to WT *S.* Tm uptake. (E) Uptake of WT and Δ*sopE2 S.* Tm into WT and ΔN-WASP MEFs or WT MEFs pretreated with 10 μM 187-1 (WT + 187-1). Values relative to uptake by WT S. Tm into WT MEFs. All *S.* Tm uptake was quantified 30 min after initial infection. Values are the means of three independent replicates (each replicate is comprised of 6 fields of view, n = 18. Each field of view is approximately 50-100 cells, see method details). Error bars indicate standard deviation. NS – no significant difference, ∗∗ - p < 0.01 (ANOVA followed by post hoc Dunnett’s comparison).

The inhibitory role of Cdc42-N-WASP is somewhat surprising as *S.* Tm delivers several effector proteins that can activate Cdc42.^27^ The *S.* Tm strain SL1344 used in this study expresses two guanine nucleotide exchange factors (GEFs): SopE1, that activates both Rac1 and Cdc42, and SopE2 that activates only Cdc42.^27^ To determine the contribution of these GEFs to Cdc42 activation during cell entry, WT MEFs were infected with SL1344 strains lacking either *sopE1*, *sopE2* or both. A GTPase activation assay determined that whilst WT or Δ*sopE1* strains effectively activated Cdc42 in the cell, Δ*sopE2* and Δ*sopE1/E2* strains did not, indicating that SopE2 is largely responsible for *S.* Tm driven activation of Cdc42 (Figure 2A). To determine whether this SopE2-dependent activation of Cdc42 resulted in increased N-WASP activation, GFP-Cdc42 was isolated from uninfected and infected cells using a GFP Trap (Chromotek) and the level of bound N-WASP measured by immunoblotting. Infection of cells with WT but not Δ*sopE2 S.* Tm resulted in a substantial increase in N-WASP binding to Cdc42 (Figure 2B), indicative of SopE2-dependent N-WASP activation. Consistent with this, ectopic expression of SopE2, but not SopE1, (Figure S2A), caused a 50% decrease in *S.* Tm uptake, which was completely reversed in the presence of the N-WASP inhibitor 187-1 (Figure 2C).

As exogenous SopE2 expression leads to N-WASP activation, we next explored how native SopE2 delivered by *S.* Tm contributes to entry. As previously demonstrated,^11^ the control deletion of s*opE1* (Δ*sopE1*) impeded invasion into WT MEFs by over 55%, yet the loss of *sopE2* (Δ*sopE2*) promoted invasion by over 25% (Figure 2D). Similar results were also observed in Hap1 cells (Figure S2B). The increase in invasion caused by the loss of SopE2 expression was not observed in Δ*N-WASP* MEFs, or WT MEFs treated with 187-1, strongly suggesting that the inability of *S.* Tm Δ*sopE2* to activate N-WASP is responsible for the increased invasion (Figure 2E). Collectively, these results indicate that activation of N-WASP by delivered SopE2 is inhibitory for *S.* Tm entry.

### SopE2 and N-WASP inhibit *Salmonella* uptake at later timepoints of invasion

**Figure 3:**
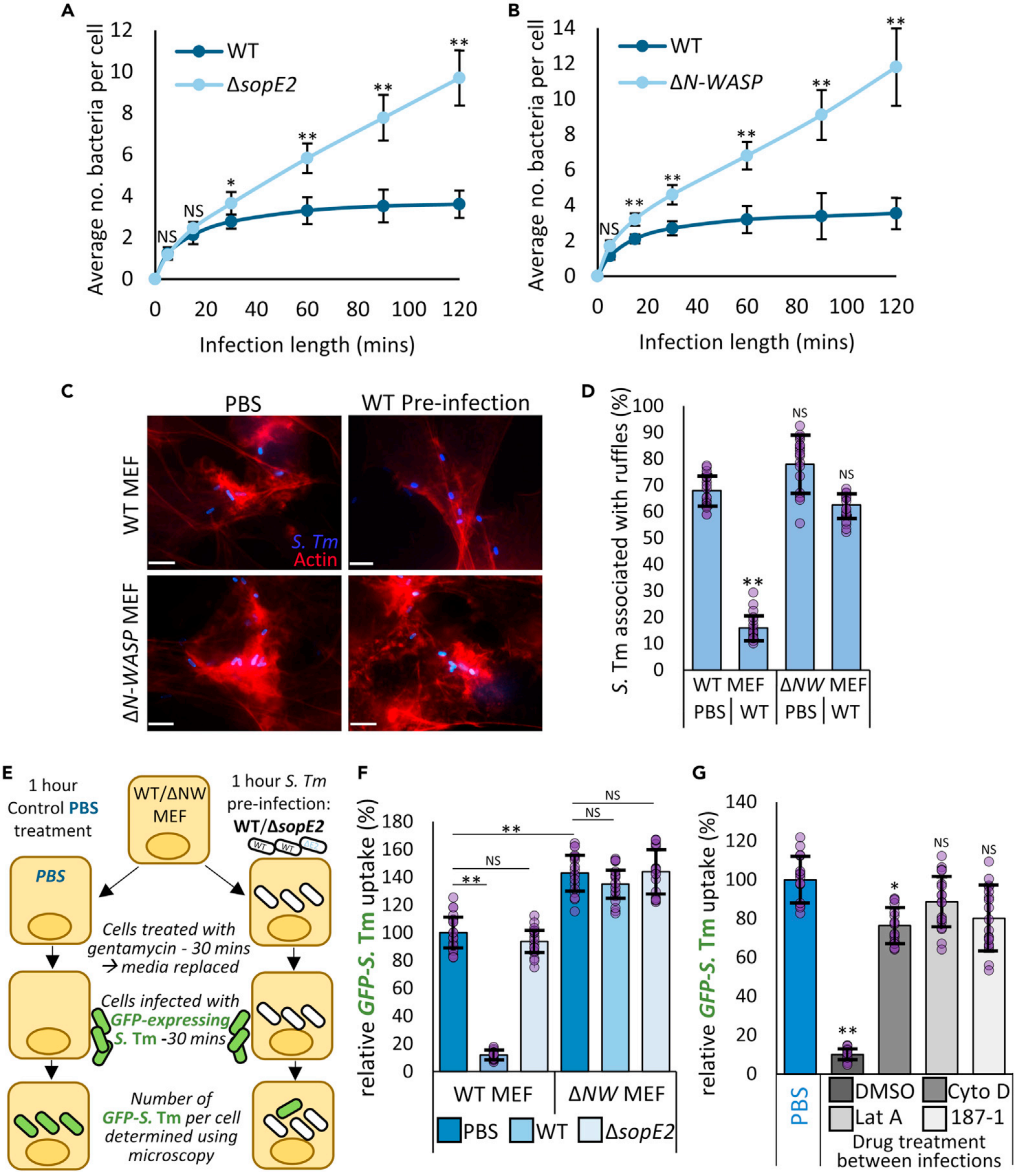
SopE2 and N-WASP inhibit *Salmonella* uptake at later timepoints of invasion (A) Average number of WT and Δ*sopE2 S.* Tm taken up into WT MEFs after indicated infection length. (B) Average number of WT *S.* Tm taken up into WT and Δ*N-WASP* MEFs after indicated infection length. (C) Fluorescence microscopy images of WT and Δ*N-WASP* MEFs pre-infected for 60 min with unstained WT *S.* Tm (WT Pre-infection) or treated with control PBS, then infected for 5 min with Alexa Fluor 350 pre-stained WT *S.* Tm (Blue). Actin was visualized with Texas Red Phalloidin (Red). Scale bar is 2 μm. (D) Average percentage of *S.* Tm associated with an actin-rich ruffle. WT or Δ*N-WASP* MEFs were pre-infected with unstained WT *S.* Tm (WT) or treated with PBS as a control (PBS), then infected for 5 min with WT *S.* Tm pre-stained with Alexa Fluor 350. Cells were stained with Texas Red phalloidin to visualise actin (Red). (E) Diagram depicting protocol for double infection experiments used in (F) and (G). (F) Uptake after 30 min of WT GFP-expressing *S.* Tm into WT and Δ*N-WASP* MEFs that had been pre-infected for 60 min with non–GFP-expressing WT or Δ*sopE2 S.* Tm, or mock treated with PBS. All values relative to WT *S.* Tm uptake into WT MEFs pretreated with PBS control. (G) Restoration of invasive capacity to pre-infected cells by drug treatment: WT MEFs were pre-infected with WT *S.* Tm not expressing GFP for 60 min, and then treated with either DMSO, 5 μM Cytochalasin D (Cyto D), 5 μM Latrunculin A (Lat A) or 10 μM 187-1 for 15 min as indicated. The drug was then washed out thoroughly, and the uptake of fresh, GFP-expressing *S.* Tm measured after 30 min. Values are relative to WT *S.* Tm uptake into WT MEFs pretreated with PBS control. All *S.* Tm uptake and ruffle formation values are the means of three independent replicates (each replicate is comprised of 6 fields of view, n = 18. Each field of view is approximately 50-100 cells, see method details). All error bars indicate standard deviation. NS – no significant difference, ∗∗ - p < 0.01, ∗ - p < 0.05 (ANOVA followed by post hoc Dunnett’s comparison).

The finding that the “entry” effector SopE2 is antagonistic to *S.* Tm invasion is somewhat unexpected. To gain a deeper insight into the role of SopE2, *S.* Tm invasion assays were performed using infection lengths ranging from 5 to 120 min with both WT and Δ*sopE2* strains (Figure 3A). After a 5- or 15-min infection there was no significant difference in invasion by WT or Δ*sopE2 S.* Tm*,* yet after 30 min there was a clear, approximately 20% increase in the entry of Δ*sopE2* as we have already demonstrated (Figure 2D). Strikingly, the uptake of WT *S.* Tm peaks between 30 and 60 min, with longer infection times leading to almost no increase in intracellular bacteria. In contrast, invasion of Δ*sopE2 S.* Tm continued to occur steadily over the entirety of the 120-min infection, resulting in an almost 2.5-fold higher number of internalized bacteria than for WT *S.* Tm. The same unrestricted invasion was observed when WT *S.* Tm infected either Δ*N-WASP* MEF (Figure 3B) or Hap1 (Figure S3A) cells, or WT MEFs treated with the N-WASP inhibitor 187-1 (Figure S3B). Together, this suggests that SopE2 activation of N-WASP limits the number of *S.* Tm that are able to enter each individual cell.

We hypothesized that in our assay, after 30-60 min *S.* Tm-delivered SopE2 is able to activate sufficient N-WASP to block further bacterial uptake from occurring. Interestingly, the characteristic membrane ruffles formed by *S.* Tm to drive invasion failed to form in WT MEFs that had been pre-infected for 1 h with unstained WT *S.* Tm. Ruffles did however form in Δ*N-WASP* MEFs that had been pre-infected, as well as in WT and Δ*N-WASP* MEFs that had been pretreated with PBS as a control (Figure 3C). In control PBS-pretreated WT and Δ*N-WASP* MEFs, and Δ*N-WASP* MEFs pre-infected with WT *S.* Tm, approximately 60-75% of fresh *S.* Tm were associated with actin-rich ruffles, with this number falling to 15% in WT MEFs pre-infected with WT *S.* Tm (Figures 3D, S3C).

To demonstrate that the inability to form membrane ruffles in pre-infected cells also affected *S.* Tm uptake, double infection invasion assays were carried out (illustrated in Figure 3E). Briefly, WT or Δ*N-WASP* MEFs were pretreated with PBS, or pre-infected with WT or Δ*sopE2 S.* Tm for 60 min, before being treated with gentamycin for 30 min to kill extracellular bacteria. Subsequently, these pre-infected cells were subjected to a second 30-min infection with fresh WT *S.* Tm that express GFP only when internalized,^28^ allowing the quantitation of their invasion. Pre-infection with WT *S.* Tm reduced subsequent invasion by fresh bacteria by almost 90%, compared to control cells, while cells that were pre-infected with Δ*sopE2 S.* Tm, were invaded similarly to control cells (Figure 3F). The ability to seemingly make the cell impervious to further rounds of *S.* Tm uptake lasts for at least 4 h after the initial infection (Figure S3D), and importantly, pre-infection of Δ*N-WASP* cells by either WT or Δ*sopE2 S.* Tm had no effect on invasion of fresh WT *S.* Tm (Figure 3F). An inhibition in the uptake of WT *S.* Tm was also clear in WT MEFs, but not Δ*N-WASP* MEFs that were pre-infected with Enteropathogenic *Escherichia coli* (EPEC), a pathogen well known to activate N-WASP for actin pedestal formation required for cellular attachment^29^ (Figure S3E).

Collectively, these data suggest that N-WASP-dependent actin assembly induced by SopE2 delivery makes cells resistant to further pathogen uptake. To test this hypothesis, we attempted to restore invasion susceptibility to pre-infected cells. To achieve this, cells pre-infected with WT *S.* Tm (as above) were treated for 15 min with either control DMSO, the N-WASP inhibitor 187-1 or the actin destabilizing drugs Cytochalasin D^30^ or Latrunculin A.^31^ The cells were then thoroughly washed to remove the drugs, and then infected with fresh WT *S.* Tm. Strikingly, the inhibition of *S.* Tm invasion observed in pre-infected cells was almost completely reversed when cells were treated with Cytochalasin D, Latrunculin A or 187-1 but not by a DMSO control (Figure 3G). This strongly implies that actin assembled by N-WASP in response to infection impedes further *S.* Tm from stimulating their own actin-dependent uptake.

### SopA controls how SopE2 affects *Salmonella* uptake

**Figure 4:**
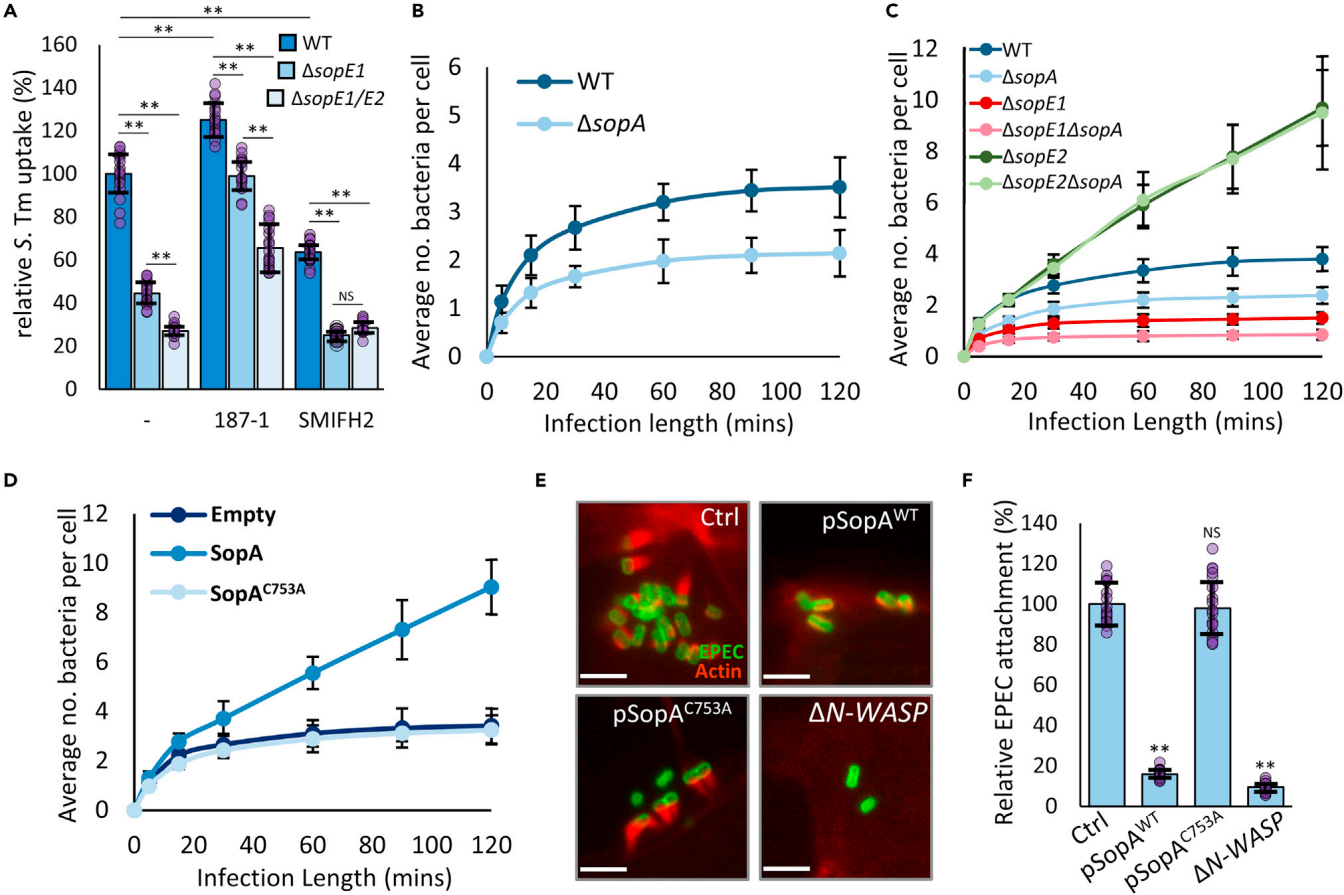
SopA controls how SopE2 affects *Salmonella* uptake (A) Uptake of WT, Δ*sopE1* and Δ*sopE1/E2 S.* Tm after 15 min infection of WT MEFs (−) or WT MEFs pretreated with 10 μM 187-1 or 10 μM of the pan-formin inhibitor SMIFH2. Values relative to WT *S. Tm* uptake into control WT MEFs. (B) Average number of WT and Δ*sopA S.* Tm taken up into WT MEFs after indicated infection length. (C) Average number of WT, Δ*sopA*, Δ*sopE1*, Δ*sopE1*Δ*sopA*, Δ*sopE2,* and Δ*sopE2*Δ*sopA S.* Tm taken up into WT MEFs after indicated infection length. (D) Average number of WT *S.* Tm taken up into WT MEFs ectopically expressing a control empty vector (Empty), SopA, or SopA^C753A^ after indicated infection length. (E) Fluorescence microscopy images of actin pedestal formation by WT EPEC (Green) after a 90-min infection in WT MEFs ectopically expressing a control empty vector (Ctrl), WT SopA (pSopA) or SopA^C753A^ (pSopA^C753A^), or in Δ*N-WASP* MEFs (Δ*N-WASP*). Texas Red Phalloidin was used to stain actin (Red). Scale bar 1.5 μm. (F) Average attachment of WT EPEC to WT MEFs WT MEFs ectopically expressing a control empty vector (Ctrl),WT SopA (pSopA^WT^) or SopA^C753A^ (pSopA^C753A^), or to Δ*N-WASP* MEFs (Δ*N-WASP*). Values relative to attachment to WT MEFs transfected with a control empty vector. All *S.* Tm uptake and EPEC attachment values are the means of three independent replicates (each replicate is comprised of 6 fields of view, n = 18. Each field of view is approximately 50-100 cells, see method details). All error bars indicate standard deviation. NS – no significant difference, ∗∗ - p < 0.01, ∗ - p < 0.05 (ANOVA followed by post hoc Dunnett’s comparison).

Our results demonstrate that SopE2 and N-WASP negatively regulate *S.* Tm uptake, but it is also apparent that this inhibition only occurs at later timepoints (30 min or later in our assay). SopE2 may therefore also play a role in promoting invasion at earlier timepoints. Consistent with this, deletion of *sopE2* in a Δ*sopE1* background results in a small additional defect in invasion after 15 min (Figure 4A). Indeed, the Δ*sopE1/E2* strain invades poorly after 5, 15 and 30 min, but at later timepoints, in the absence of the inhibitory SopE2-Cdc42-N-WASP pathway, internalization of *S.* Tm continues to occur, eventually overtaking that seen for WT *S.* Tm (Figure S4A). This indicates that at early stages of infection SopE2 plays a small but positive role in invasion, an effect likely masked in strains where invasion is driven by the dominant SopE1. Cdc42 activated by SopE2 may promote this early invasion via N-WASP, or via other proteins activated by Cdc42 that drive actin assembly - such as formins (known to play a role in *S.* Tm uptake^32^). To test this hypothesis MEFs treated with 187-1 or the pan-formin inhibitor SMIFH2^33^ were infected with WT, Δ*sopE1*, and Δ*sopE1/E2 S.* Tm (Figure 4A). N-WASP inhibition promoted invasion by all three *S.* Tm strains at 15 min. Interestingly however, both the Δ*sopE1* and Δ*sopE1/E2* strains invade to a similar extent in the presence of SMIFH2, indicating that in the absence of SopE1 at least, SopE2 activation of Cdc42 can promote invasion in a formin-dependent manner at early stages of an infection.

SopE2 therefore may play both a positive and negative role in *S.* Tm cell invasion. SopE2 is found in almost all serovars of *S. enterica*,^34^ and its sequence is highly conserved (Figure S4B), yet in serovar Typhi (*S*. Typhi) it is pseudogenised.^35^ Consistent with our data here, the introduction of SopE2 from *S.* Tm into *S.* Typhi greatly impedes the uptake of *S*. Typhi into cultured cells.^35^ More intriguingly this inhibition could be overcome through the expression of *S*. Tm SopA, a second effector which is also pseudogenised in *S*. Typhi.^35^ This suggests that SopA may be able to prevent SopE2 from inhibiting *S*. Tm uptake and thus function to control the balance of positive and negative contributions of SopE2 to invasion.

To investigate this, we performed 5-120-min infection time-course experiments. The loss of SopA (Δ*sopA*) caused a modest, statistically significant, inhibition of invasion (15-35%) at all timepoints measured (Figure 4B), consistent with previous reports.^36^ The Δ*sopE1* strain invades more poorly than WT at all timepoints (55% less at 30 min) and like WT invasion does not continue after 60 min (Figure 4C). The additional deletion of *sopA* (Δ*sopE1*Δ*sopA*) results in a further drop in invasion at all timepoints relative to Δ*sopE1* (Figure 4C, the two strains plotted alone for clarity in Figure S4C). However, the deletion of *sopA* in the strain lacking *sopE2* (Δ*sopE2*Δ*sopA)* did not have a negative effect on invasion (Figure 4C), indicating that SopE2 is responsible for the entry defect observed in the other strains lacking SopA. The invasion of Δ*sopE1*Δ*sopA* after 15 min is almost identical to that of ΔsopE1/E2 (Figure S4D) which suggests that the positive contribution of SopE2 to uptake at early stages of invasion (Figure 4A) is dependent on the presence of SopA. Therefore, in the absence of SopA, Cdc42-N-WASP signaling, driven by SopE2, is unimpeded, causing invasion to be inhibited, and any positive role for SopE2 at early stages is lost.

Consistent with the idea that SopA prevents Cdc42-N-WASP signaling from inhibiting invasion, WT *S*. Tm invasion of WT MEFs ectopically expressing SopA (Figure S4E) continued to occur steadily 30-120 min after the start of infection (Figure 4D), as seen above for Δ*sopE2* entry (Figure 3A). Ectopic expression of SopA also resulted in the continuous invasion of Δ*sopA S*. Tm, as well as the continued uptake of the poorly invasive Δ*sopE1*. SopA expression did not have an additional effect on the already continuously invasive Δ*sopE2* strain (Figure S4F). SopA is a ubiquitin ligase,^37^ and expression of an inactive variant (SopA^C753A^) did not affect the uptake of WT *S*. Tm (Figure 4D), suggesting that ubiquitination likely regulates SopE2 signaling.

Enteropathogenic. *E. coli* (EPEC) form actin pedestals and tightly adhere to target cells in an N-WASP-dependent manner.^29^ In cells expressing SopA, but not SopA^C753A^, EPEC pedestal formation was greatly compromised (Figure 4E), with extremely small or completely absent pedestals. These effects were mirrored by the overall ability of EPEC to attach to cells, with attachment to cells expressing SopA being similar to that to Δ*N-WASP* cells (reduced by 83% and 88% respectively; Figure 4F, with representative images in Figure S4G). This data rules out the possibility that SopA interferes directly with SopE2, and instead, SopA must modulate the cellular Cdc42-N-WASP pathway, either directly or indirectly.

Together the data presented here demonstrate that the Cdc42-N-WASP pathway, activated by SopE2, is able to block *S.* Tm uptake into host cells. During early periods of invasion SopA, in a ubiquitin ligase-dependent manner, is able to prevent this by interfering with Cdc42-N-WASP signaling, thus allowing *S.* Tm to efficiently invade cells.

## Discussion

**Figure 5:**
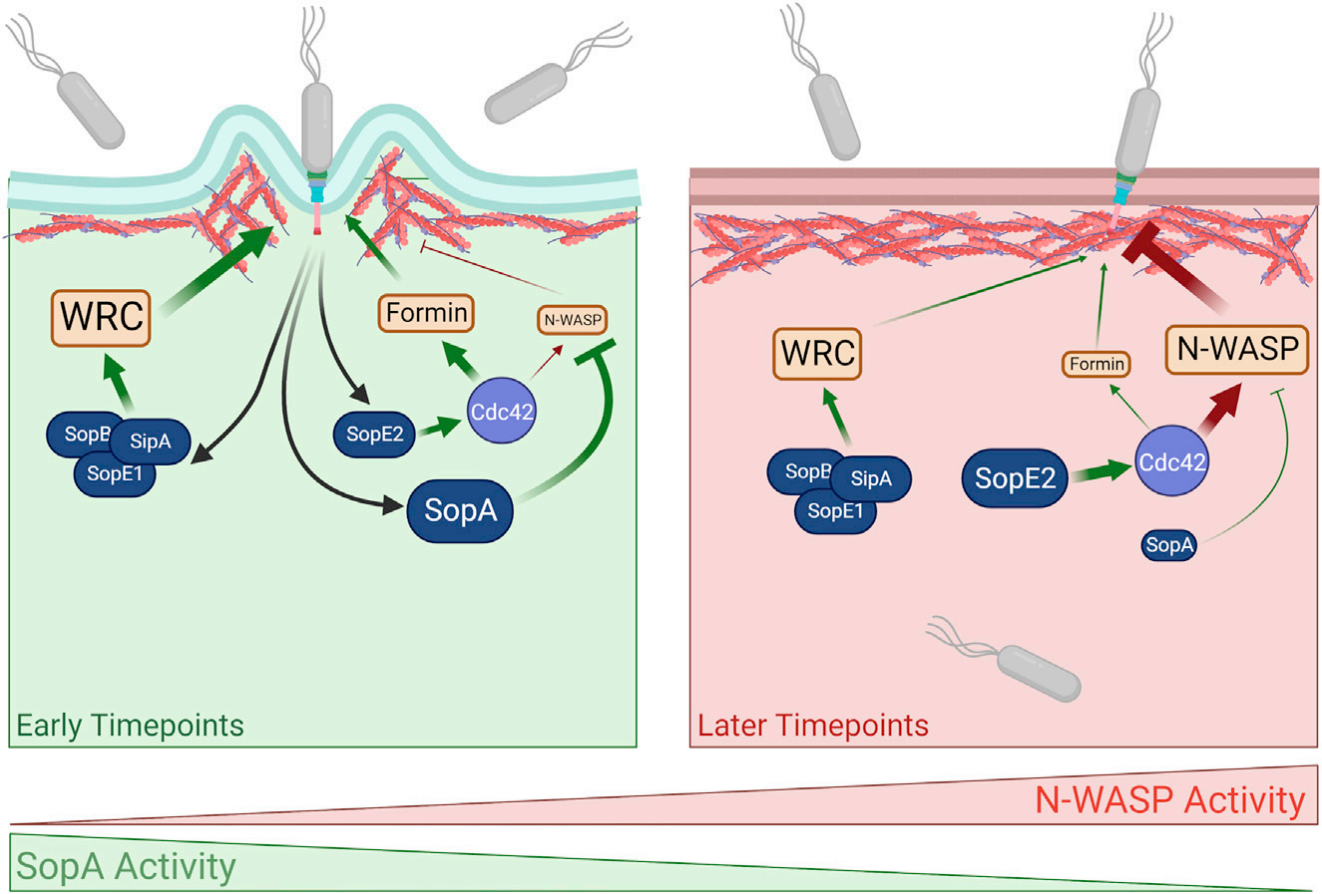
SopA and N-WASP activity determine host cell susceptibility to *Salmonella* uptake At early timepoints during infection *S.* Tm readily enter host cells (left). Uptake is driven by effectors such as SopE1, SopB, and SipA that remodel the actin cytoskeleton. SopE2 is unable to activate sufficient N-WASP to impede *S.* Tm invasion, and this is likely due to high levels of SopA activity. Instead, SopE2 may promote uptake, activating Cdc42 that in turn could potentially activate formins. At later timepoints, cells become recalcitrant to *S.* Tm entry (right). As SopA activity decreases, SopE2-driven N-WASP activation increases, which in turn impedes the actin turnover that drives *S.* Tm uptake. Created with BioRender.com.

The data here allow us to propose a model for how the host cell’s susceptibility to invasion by *S.* Tm is determined by the Cdc42-N-WASP pathway (Figure 5). We postulate that early during invasion, N-WASP activity is low, and the host cell is susceptible to efficient *S.* Tm uptake. We observe that, on average, 2-3 bacteria enter per cell during this phase in our assay conditions. At later timepoints, as N-WASP activity increases the cell becomes recalcitrant to further *S.* Tm uptake, and invasion is effectively blocked. This inhibition of *S.* Tm uptake is driven by the effector SopE2, which activates the Cdc42-N-WASP pathway. At early timepoints, SopE2-dependent activation of Cdc42 does not inhibit *S.* Tm uptake and instead, Cdc42 plays a minor role in promoting uptake, potentially via activation of formins (Figure 4). The transition of a host cell from being susceptible to *S.* Tm invasion to a state that is almost totally recalcitrant to uptake is controlled by the activity of the *S.* Tm effector SopA. At early timepoints, SopA activity is high, and N-WASP activity is consequently low, despite the activation of Cdc42 by SopE2, meaning the host cell can be invaded. As the activity of SopA is diminished, and N-WASP activity increases due to the action of SopE2, a threshold is met at which point the cell becomes impervious to further *S.* Tm uptake. In other words, although SopE2 activates Cdc42 at all phases of entry, this only triggers N-WASP-dependent actin assembly (and consequent inhibition of further uptake) at later timepoints, i.e. when SopA activity is reduced.

We demonstrate throughout this work that the activation of N-WASP, and subsequent N-WASP driven actin assembly, is inhibitory to *S.* Tm-driven uptake into host cells. It is likely that the precise level of Cdc42/N-WASP activity required to prevent pathogen uptake, and the time taken to achieve this level during infection will vary between different cell types and/or culture conditions. As N-WASP triggers actin assembly and is recruited to *S.* Tm entry foci,^20^ it is generally assumed that N-WASP, and its activation by SopE2, positively contributes to invasion.^20^^,^^38^^,^^39^ The only direct evidence for this is one study showing that invasion is inhibited by expression of a “dominant-negative” N-WASP construct, however this construct non-productively binds Cdc42, and thus would inhibit all Cdc42-dependent signaling, not just that to N-WASP.^20^ More recently, and consistent with our results, it has been reported that *S.* Tm entry into N-WASP knockout cells is actually enhanced compared to wild-type cells.^18^

Determining precisely why N-WASP-driven actin assembly is inhibitory to *S.* Tm forced uptake will require further study. It is logical that the activation of N-WASP and generation of actin filaments would result in a competition for resources, such as the Arp2/3 complex, capping proteins, cofilin, and free actin. Additionally, N-WASP is well-known to play a role in clathrin-mediated endocytosis^40^ and could potentially recycle important actin machinery away from the plasma membrane. The actin generated by N-WASP activity may also act as a physical barrier that blocks other forms of actin assembly, or indeed the efficient delivery of effectors from extracellular *S.* Tm. Indeed, cortical actin assembly increases cell rigidity, which in turn can restrict the uptake of *S.* Tm into macrophages,^41^ and *Pseudomonas aeruginosa* into lung epithelial cells.^42^ It has also been reported that WASP, the Dictyostelium *N-WASP homolog,* is able to restrict Rac1 activity,^43^ and it is well established that Rac1 is a key driver of *S.* Tm uptake.^44^ Many other invasive bacteria and viruses activate N-WASP during the course of their own infection of host cells^45^ and it will be intriguing to explore the role of N-WASP in these systems.

Unlike its homolog SopE1, whose role in promoting *S.* Tm uptake is well-established, the contribution of SopE2 to entry is less clear. Deletion of *sopE2* in strains that express SopE1, e.g. SL1344 used here, has little effect on *S.* Tm entry.^22^ Likewise, Cdc42, the target of SopE2’s GEF activity, is not required for *S.* Tm uptake.^44^^,^^46^ In the absence of *sopE1*, deletion of *sopE2* has a more pronounced negative impact,^34^ and in strains engineered to lack multiple effector proteins, expression of SopE2 alone is able to restore invasion of various cultured cells to approximately 10-20% of WT.^38^ In contrast, the introduction of *S.* Tm *sopE2* into *S. Typhi*, in which *sopE2* is pseudogenised, results in reduced uptake of the bacteria.^35^ Recent work has demonstrated that, in a strain lacking SopE1, both SopE2 and the actin-binding effector SipA play key, but redundant, roles in *S.* Tm invasion of the intestinal epithelium of neonate mice.^47^ However, in adult mice invasion (by a *sopE1* positive strain) can be severely reduced by the deletion of *sipA* alone, suggesting that SopE2 plays a less significant role.^48^ Consistent with all these findings, our data show that SopE2 activation of Cdc42 has a small, N-WASP-independent, contribution to invasion at early timepoints, and that this is more evident in strains lacking *sopE1* (Figure S4). We propose therefore that Cdc42 activation by SopE2 initially promotes *S.* Tm uptake to varying degrees in a strain-, cell type- and/or developmental stage-dependent manner. Our data suggest this may be due to the activation of formins (Figure 4), but it could also potentially involve further downstream substrates of Cdc42, such as p21-activated kinase.^49^ Eventually however, the activation of Cdc42-N-WASP by SopE2 reaches a threshold that leads to the cell becoming recalcitrant to further bacterial uptake.

While we can’t rule out a role for changes in SopE2 level and/or localization,^50^ it seems likely that the transition of Cdc42 from promoter to inhibitor of invasion is controlled by SopA as in the absence of SopA the small positive contribution of SopE2-Cdc42 at early stages of invasion isn’t evident (Figure S4C), whereas in cells ectopically over-expressing SopA the N-WASP-dependent inhibition of uptake is prevented (Figure 4). This suggests that the transition to the dominant inhibitory N-WASP activity at later timepoints of WT infection could be linked to a loss of SopA activity in the cell. SopA is a ubiquitin ligase that can both auto-ubiquitinate and be a substrate for the host ubiquitin ligase RMA1^51^^,^^52^ both of which may contribute to degradative ubiquitination which has been observed during infection.^53^ In addition, the small RNA IsrM can repress SopA expression,^54^ and is responsible for turning off SopA production at later stages of *S.* Tm infection of murine macrophages.^55^ SopA levels are thus tightly controlled both pre- and post-translocation into host cells. It is also possible that the changes in SopA activity seen at later timepoints are due to altered SopA localization. The precise dynamics of SopA activity in the cell and how this correlates with the invasion potential of cells therefore requires further study, as does the precise mechanism by which SopA regulates signaling downstream of SopE2. This requires its ubiquitin ligase activity (Figure 4D), and as SopA can also inhibit EPEC attachment (Figures 4F and 4G) its target is not SopE2 itself but a downstream cellular factor such as Cdc42 or N-WASP. The most prominent host substrates for SopA identified from proteomic studies are themselves ubiquitin ligases, namely TRIM56 and TRIM65, and targeting of these allows *S.* Tm to modulate the host inflammatory response.^56^^,^^57^ Neither TRIM56 nor TRIM65 have any known link to Cdc42-N-WASP signaling or any other cytoskeletal pathway, so are likely not responsible for the effects described here. In the specific cell line and conditions assessed, many other proteins showed low-level SopA-dependent ubiquitination, including Cdc42 and N-WASP.^57^ Intriguingly, a global study of host proteins ubiquitinated in response to *S.* Tm infection showed that Cdc42 is highly ubiquitinated at early timepoints (30 min), though the ubiquitin ligase responsible was not identified.^53^

Irrespective of the precise mechanism, the idea that an intracellular pathogen such as *S.* Tm would restrict its own uptake is at first glance surprising. However, for many viruses, including HIV,^58^ hepatitis C^59^ and influenza^60^ once a cell is infected, further infection is inhibited, a phenomenon termed superinfection exclusion. A similar mechanism has been described for the intracellular bacterial pathogen Chlamydia^61^ and presumably may ensure maximal resources for replication for the initially invading pathogens. Interestingly, in models of systemic infection in mice, sites of *S.* Tm colonization such as the spleen and gall bladder generally only contain one or two bacteria per cell.^62^^,^^63^ In humans *S.* Tm usually causes gastroenteritis and doesn’t spread systemically.^3^ *S.* Tm gastroenteritis is characterized by a localized inflammatory response triggered by the pathogen invading cells of the intestinal epithelium. However, the majority of the colonizing *S.* Tm remain in the lumen of the intestine where they take advantage of the inflammatory response to outcompete resident microbiota and spread to new hosts via the faeco-oral route.^5^^,^^6^ Suppressing entry into already-infected cells may result in a greater proportion of infected cells, thereby amplifying the triggered immune response. In this scenario, it also makes sense that once a sufficient inflammatory response has been triggered, *S.* Tm has a mechanism to limit further unnecessary invasion, especially as the subpopulation that invade host cells are eventually killed, presumably by host immune cells such as neutrophils. In contrast to *S.* Tm, the disease caused by the human-adapted *S.* typhi (i.e. typhoid fever) is non-inflammatory—following entry into intestinal epithelium cells, the pathogen spreads systemically to colonize sites such as the gall bladder, from where the bacteria can be secreted into intestinal lumen and subsequently spread to new hosts.^64^ Provocatively, both *sopE2* and *sopA* have been pseudogenized in *S. typhi*,^35^ suggesting that following the evolution of a systemic lifestyle this pathogen no longer requires a mechanism to limit cell invasion.

In conclusion, we show that rather than driving the entry of *Salmonella* into non-phagocytic cells, the Cdc42-N-WASP pathway, activated by the delivered effector SopE2, is instead inhibitory to pathogen uptake. N-WASP activation has been implicated in the invasion of host cells by many intracellular bacterial pathogens,^45^ though as is the case for *S.* Tm, the direct evidence for this is often lacking. In fact, in at least one of these cases, namely *Shigella flexneri*, it has also been reported that exogenous expression of N-WASP is inhibitory to pathogen entry,^26^ as we describe here for *Salmonella*. This raises the possibility that the specific actin assembly triggered by N-WASP causes host cells to become resistant to invasion by multiple bacterial pathogens. The manipulation of N-WASP signaling by various bacterial species, and how this contributes to pathogenesis, must therefore be carefully re-assessed.

### Limitations of the study

We have shown that the effector-driven activation of N-WASP during *S.* Tm invasion of cells prevents the entry of further bacteria. We do not yet know how N-WASP is inhibitory, but this inhibition is apparent in multiple different cell types. Our data suggest that SopA supresses the inhibitory N-WASP pathway to allow *S*. Tm to enter cells at early timepoints, and we hypothesize that SopA activity is later diminished. However, as our antibodies are unable to detect the low levels of SopA delivered during infection we do not know if this is due to degradation or altered expression or localization. We cannot therefore model how this activity of SopA coordinates with its other reported function in modulating the inflammatory response. Cell culture assays allow easy genetic manipulation of target cells, they also allow the study of cell entry with high temporal resolution and in isolation from factors such as the host immune system and competing microbiota. This is especially important when studying multifunctional effectors such as SopE2 and SopA which also play a role in modulating the host immune response to infection. However, understanding the importance of N-WASP-driven entry restriction, and its modulation by SopA, to disease pathogenesis will ultimately require *in vivo* studies.

## STAR★Methods

### Key resources table

**Table:** 

REAGENT or RESOURCE	SOURCE	IDENTIFIER
**Antibodies**		

Rabbit anti-GFP	Chromotek	Cat# 3h9, RRID:AB_10773374
Mouse anti-Tubulin	Abcam	Cat# ab7291, RRID:AB_2241126
Rabbit anti-Arp2	Abcam	Cat# ab129018, RRID:AB_11157898
Mouse anti-Cdc42	Santa Cruz Biotechnology	Cat# sc-8401, RRID:AB_627233
Mouse anti-Rac1,2,3	Santa Cruz Biotechnology	Cat# Sc-514583, RRID:AB_2818941
Rabbit anti-Nap1	Sigma-Aldrich	Cat# N3788, RRID:AB_1841025
Rabbit anti-Actin	Sigma-Aldrich	Cat# A2066, RRID:AB_476693
Rabbit anti-N-WASP	Diagnostics Scotland	N/A
Rabbit anti-SopA	Diagnostics Scotland	N/A
Rabbit anti-intimin	Diagnostics Scotland	N/A
IRDye680 RD Donkey anti-Rabbit IgG	LI-COR Biosciences	Cat# 926–68073, RRID:AB_10954442
IRDye 800CW Goat anti-Mouse IgG	LI-COR Biosciences	Cat# 926–32210, RRID:AB_621842

**Bacterial and virus strains**		

*S.* Tm (*Salmonella enterica* serovar Typhimurium, SL1344)	Gift from Jean Guard-Petter	N/A
*S.* Tm Δ*sopE1*	Gift from Wolf Dietrich-Hardt	N/A
*S.* Tm Δ*sopE1/E2*	Gift from Wolf Dietrich-Hardt	N/A
*S.* Tm Δ*sopE2*	This paper	N/A
*S.* Tm Δ*sopA*	This paper	N/A
*S.* Tm Δ*sopE1*Δ*sopA*	This paper	N/A
*S.* Tm Δ*sopE2*Δ*sopA*	This paper	N/A
*Escherichia coli* 0127:H6 (Strain E2348/69 EPEC)	Gift from John Leong	N/A

**Chemicals, peptides, and recombinant proteins**		

187-1	Tocris Bioscience	Cat# 2061
SMIFH2	Sigma-Aldrich	Cat# S4826
Latrunculin A	Cayman Chemicals	Cat# 10010630
Cytochalasin D	Sigma-Aldrich	Cat# C2618
DMSO	Thermo-Fisher	Cat# TS-20684
Protease inhibitor Cocktail	Sigma-Aldrich	Cat# P8340
Texas-Red Phalloidin	Thermo-Fisher	Cat# T7471
RIPA Buffer	Sigma-Aldrich	Cat# R0278
Glutathione Sepharose 4B	Merck	Cat# GE17-0756-01
Alexa-Fluor 350 Carboxylic Acid Succinimidyl ester	Thermo-Fisher	Cat# A10168
GFP Trap Agarose	Chromotek	Cat# gta

**Deposited data**		

Raw data for generating graphs, raw western blots and PH-VCA sequence.	Mendeley Data repository	https://doi.org/10.17632/dhh6mbjky5.1

**Experimental Models: Cell Lines**		

WT MEF	Gift from Leszek Kotula^65^	N/A
Δ*N-WASP* MEF	Gift from Scott Snapper^66^	N/A
Δ*Abi1* MEF	Gift from Leszek Kotula^65^	N/A
WT Hap1	Horizon Discovery	Cat# C061, RRID:CVCL_Y019
Δ*N-WASP* Hap1	Horizon Discovery	Cat# HZGHC002632c011, RRID:CVCL_TX84
Δ*Nap1* Hap1	Horizon Discovery	Cat# HZGHC003401c001, RRID:CVCL_TA06
HeLa	Merck	Cat# 93021013, RRID:CVCL_0030
CaCo-2	Sigma-Aldrich	Cat# 09042001, RRID:CVCL_0025

**Oligonucleotides**		

Oligonucleotides	See Table S1 for a list of all oligonucleotides.	N/A

**Recombinant DNA**		

**pEmerald-N-WASP***For ectopic expression of Emerald-N-WASP*	This paper	N/A
**pEmerald-N-WASP**^**L299P**^*For ectopic expression of Emerald-N-WASP*^*L299P*^	This paper	N/A
**pEmerald-PH-VCA***For ectopic expression of Emerald-PH-VCA*	This paper	N/A
**pEmerald-Cdc42***For Ectopic expression of Emerald GFP-Cdc42*	This paper	N/A
**pEmerald-Cdc42**^**Q61L**^*For Ectopic expression of Emerald GFP-Cdc42*^*Q61L*^	This paper	N/A
**pEmerald-Rac1**^**Q61L**^*For Ectopic expression of Emerald GFP-Rac1*^*Q61L*^	This paper	N/A
pEmerald-SopE1^78-241^*For Ectopic expression of Emerald GFP-SopE1*^*78-*^*^241^ (SopE1 without its translocation domain)*	This paper	N/A
**pEmerald-SopE2**^**70-**^**^24^***For Ectopic expression of Emerald GFP-SopE1*^*70-*^*^240^ (SopE2 without its translocation domain)*	This paper	N/A
**pEmerald***Control empty pEmerald vector*	This paper	N/A
**pHA-SopA**^**60-782**^*For Ectopic expression of HA-SopA*^60-782^*(SopA without its translocation domain)*	This paper	N/A
**pHA-SopA**^**60-782, C753A**^*For Ectopic expression of HA-SopA (SopA*^*C753A*^*without its translocation domain)*	This paper	N/A
**pHA***Control empty pHA vector*	This paper	N/A
**pM975***Expression of GFP from the SPI2 promoter allowing for detection of internalised S.Tm.*	Gift from Wolf Dietrich-Hardt^28^	N/A
**pKD13***Template for kanamycin resistance cassette for λ Red recombination*	Gift from Barry Wanner^67^	N/A
**pCP20***Expression of FLP recombinase*	Gift from Wilfried Wackernagel^68^	N/A
**pWRG717***Template for kanamycin resistance cassette for λ Red recombination*	Gift from Roman Gerlach^69^	N/A

**Software and algorithms**		

Volocity 3D Image Analysis Software	Perkin Elmer	RRID:SCR_002668
LI-COR Image Studio Software	LI-COR Biosciences	RRID:SCR_015795
Biorender	www.biorender.com	RRID:SCR_018361

### Resource availability

#### Lead contact

Further information and requests for resources and reagents should be directed to and will be fulfilled by the lead contact, Vassilis Koronakis (vk103@cam.ac.uk).

#### Materials availability

All newly generated plasmids and bacterial strains associated with this paper are available by contacting the lead contact.

### Experimental models and subject details

#### Mammalian cell culture

Mammalian cell lines are outlined in the table above. Hap1 cells (Horizon discovery) were maintained in Iscoves’s modified Dulbecco’s medium (IMDM) supplemented with 10 % Fetal Bovine Serum (FBS) and 100 U/ml penicillin-streptomycin. MEF, Caco-2 (Merck) and HeLa cells (Sigma-Aldrich) were maintained in Dulbecco’s modified Eagle’s medium (DMEM) with 10 % FBS and 100 U/ml penicillin-streptomycin. All cells were incubated at 37°C with 5 % CO_2_. Unless indicated otherwise chemical inhibitors were incubated with cells 30 minutes prior to the start of an assay at the following final concentrations: 187-1 (10 μM), SMIFH2 (10 μM), Latrunculin A (5 μM) and Cytochalasin D (5 μM). Where indicated, cells were transfected using the Neon System (Invitrogen) following the manufacturer’s instructions, 24 hours before being used in assays.

#### Bacterial strains

WT *Salmonella enterica* serovar Typhimurium SL1344 *(S.* Tm*)* was a gift from Jean-Guard Petter, and the isogenic *S.* Tm Δ*sopE1* and Δ*sopE1/E2* were gifts from Wolf-Dietrich Hardt. *S.* Tm Δ*sopE2*, Δ*sopA*, Δ*sopE1*Δs*opA* and Δ*sopE2*Δ*sopA* were generated for this paper, and details are listed below. WT *Escherichia coli* 0127:H6 (Strain E2348/69 EPEC) was a gift from John Leong.

### Method details

#### Construction of strains

*S.* Tm Δ*sopE2* was constructed by amplification of the kanamycin resistance cassette in pKD13 with primers P1 and P2 and then introducing the product into *S.* Tm using the λ Red recombinase system.^67^ Following selection on kanamycin plates, the kanamycin resistance cassette was removed by expression of FLP recombinase from pCP20.^68^ Introduction of the kanamycin cassette and its subsequent removal was confirmed by PCR of the *sopE2* gene locus with primers P3 and P4.

Strain *S*. Tm Δ*sopA* was created by first amplifying the kanamycin cassette in pWRG717 with primers P5 and P6. The recombination method described by Hoffman et al.^69^ was then used to introduce the kanamycin cassette at the *sopA* locus, verified by screening with primers P7 and P8. *S.* Tm Δ*sopA*Δ*sopE2* was created by performing the same method on the unmarked *S.* Tm Δ*sopE2* strain.

To create the *S.* Tm Δ*sopA*Δ*sopE1* strain, an unmarked deletion of *sopE1* was first created with the λ Red recombinase system using primers P9 and P10 to amplify the pKD13 kanamycin resistance cassette and primers P11 and P12 to verify its introduction at the *sopE1* gene locus. Following FLP recombinase mediated removal of the kanamycin resistance cassette, *sopA* was then deleted from the unmarked *S.* Tm *ΔsopE1* using the method described for the *S.* Tm Δ*sopA* strain.

#### Plasmids

Plasmids are outlined in the key resources table. Emerald-GFP plasmids pEmerald-N-WASP, -Cdc42, -Cdc42^Q61L^ and -Rac1^Q61L^ were generated using Gateway methodology (Invitrogen). pEmerald-N-WASP^L299P^ was generated using site-directed mutagenesis (SDM) and correct DNA mutagenesis confirmed via sequencing. DNA encoding PH-VCA was synthesised (IDT Technologies) and cloned into pEmerald using gateway methodology (sequence deposited on Mendeley: https://doi.org/10.17632/dhh6mbjky5.1). In the resulting construct, the VCA domain of N-WASP is fused to the PH domain of the Arf GEF ARNO, allowing for localisation to phosphatidylinsositol-3,4,5-triphosphate (PIP3) and PI4,5P2 that are found on the plasma membrane at *Salmonella* entry foci.^14^ pEmerald-SopE1, -SopE2, and pHA-SopA, were generated by amplification of the relevant gene from *Salmonella* SL1344 chromosomal DNA and cloning them into HA or Emerald-GFP vectors using Gateway methodology. HA-SopA^C573A^ was generated using SDM of HA-SopA and confirmed via DNA sequencing.

#### *Salmonella* infection assays

Invasion was quantified using *S.* Tm carrying the pM975 plasmid. Once internalised these bacteria express GFP via a SPI2 promoter allowing for specific detection of internalised bacteria only.^28^ Unless stated otherwise, cells were infected for 30 minutes at a multiplicity of infection (MOI) of 100, cells were washed twice in phosphate buffered saline (PBS), then incubated in media containing gentamycin (100 μg/ml) for 90 minutes. Cells were subsequently fixed, stained with Texas-Red Phalloidin (Life-Tech) and DAPI, then visualised using wide-field fluorescence microscopy with an Olympus IX81 inverted microscope. Using a 20x objective six fields of view per experimental repeat were utilised, with each field of view containing approximately 50-100 cells. The number of GFP-positive (internalised) bacteria and total number of cells (as determined from DAPI staining) per field of view were counted and the average number of bacteria per cell determined. All experiments were carried out a minimum of three times, the means calculated, and significance determined, with a P value of <0.05 (determined by ANOVA followed by a post hoc Dunnett’s comparison) deemed significant.

Double infections were carried out by first infecting cells with *S.* Tm that did not harbour the pM975 plasmid for 60 minutes (or PBS as a control). Cells were then washed in PBS and incubated in media containing gentamycin for 30 minutes, washed again and media replaced. These cells were subsequently infected for 30 minutes with *S.* Tm carrying pM975 and an invasion assay carried out and quantified as described above. For drug washout experiments, cells were incubated for 15 minutes in media containing the indicated inhibitor after the first 60-minute infection. Cells were then washed three times in PBS, media was replaced, and the second infection carried out as above.

To investigate ruffle formation, cells were infected for 5 minutes with *S.* Tm that had been pre-stained with Alexa-Fluor-350 carboxylic acid succinimidyl ester (15 minutes), then washed in Tris-buffered saline (pH 7.4). Cells were stained with phalloidin to visualise actin, then analysed by wide-field fluorescence microscopy.

#### EPEC attachment assays

MEFs were infected with WT EPEC for 90 minutes, cells were then washed twice in PBS, twice in ice cold 200 mM glycine (pH 2), then again in PBS twice. Cells were fixed, stained with Texas-Red Phalloidin and DAPI, and adherent EPEC stained using an anti-intimin antibody. The number of adherent EPEC per cell were then counted using fluorescence microscopy (of at least 500 cells). All experiments were carried out at least three times, means calculated, and significance determined by ANOVA followed by a post hoc Dunnett’s comparison (P <0.05 deemed significant).

#### GFP trap

MEFs were transfected to express GFP-tagged constructs 48 hours prior to performing the GFP trap, which was carried out according to the manufacturer’s instructions (Chromotek). The resulting trapped proteins were analysed by SDS-PAGE before being transferred to PVDF membranes and probed with appropriate antibodies. All immunoblots were visualised using a LI-COR Odyssey Fc imaging system, utilising appropriate fluorescent secondary antibodies (LI-COR).

#### GTPase activation assay

Indicated cells were washed and lysed using RIPA buffer supplemented protease inhibitors . Cell lysates were clarified by centrifugation (13,000 g, 5 minutes) then incubated with GST-PBD (that specifically and strongly interacts with GTP-bound Rac1 and Cdc42) bound to glutathione-Sepharose resin at 4°C for 30 minutes. The resin was extensively washed with PBS and recruited proteins eluted with SDS-PAGE loading buffer supplemented with 6 M urea. Eluted (and therefore active) Cdc42 was detected using SDS-PAGE and immunoblotting.

#### Sequence alignment

Sequence alignment of SopE2 proteins from multiple *Salmonella* strains was generated using Clustal Omega.^70^ Conservation was assessed in Jalview^71^.

### Quantification and statistical analysis

All *Salmonella* invasion, ruffle formation and EPEC attachment assays were performed three times independently. Statistical significance was determined using ANOVA followed by a post hoc Dunnett’s comparison. All statistical details for the experiments are provided in the figure legends, with a p < 0.05 being deemed significant. Significance is signified as follows p < 0.05 (∗), p < 0.01 (∗∗), p > 0.05 (NS).

## Data Availability

• All data is available within the paper or supplemental information. Raw western blot images, and raw data used to generate bar charts has been deposited at Mendeley and are publicly available as of the date of publication: https://doi.org/10.17632/dhh6mbjky5.1. • This paper does not report original code. • Any additional information required to reanalyse the data reported in this paper is available from the lead contact upon request.
