# Supplementary material for: Salmonella invasion of a cell is self-limiting due to effector-driven activation of N-WASP
Source: iScience. 2023 Apr 12;26(5):106643. doi: 10.1016/j.isci.2023.106643 (PMC10164908; doi:10.1016/j.isci.2023.106643)
Supplement: Document S1. Figures S1–S4 and Table S1 [file mmc1.pdf]

iScience, Volume *26*

Supplemental information

Salmonella invasion of a cell is self-limiting due to effector-driven activation of N-WASP

Anthony Davidson, Peter J. Hume, Nicholas P. Greene, and Vassilis Koronakis

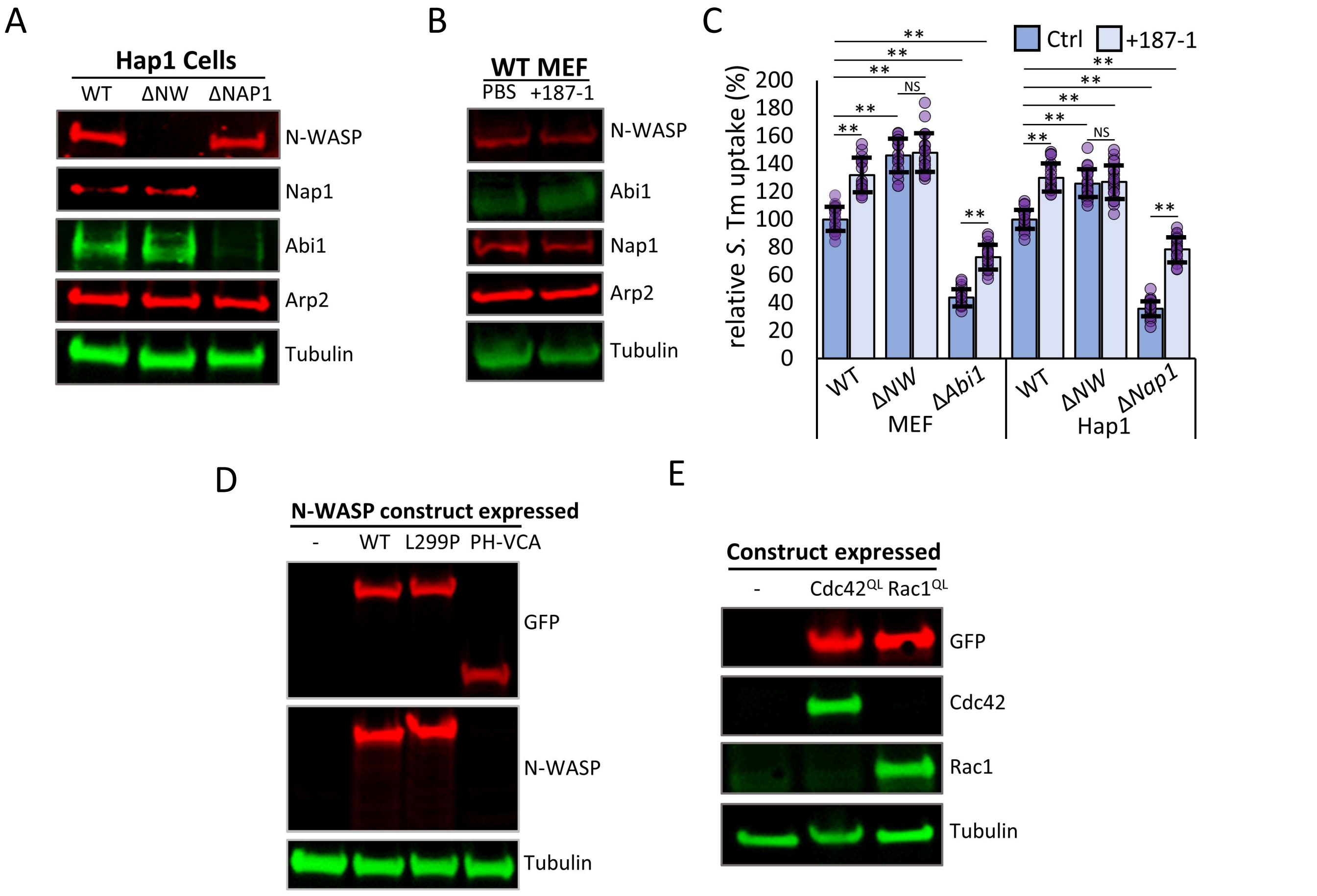

Figure S1: N-WASP inhibits *Salmonella* uptake. Related to Figure 1. (A) Immunoblot of WT, Δ*N-WASP* (Δ*NW*) and Δ*Nap1* Hap1 cell lysates depicting relative levels of N-WASP, Nap1, Abi1, Arp2 and loading control tubulin. **(B)** Immunoblot of cell lysates from MEFs pretreated with either control PBS or 10 µM 187-1 (+187-1), depicting relative levels of N-WASP, Abi1, Nap1, Arp2 and loading control tubulin. **(C)** Uptake of WT *S.* Tm (30 minutes) into WT, ΔN-WASP (ΔNW) and ΔAbi1 MEFs, and WT ΔN-WASP and ΔNap1 Hap1 cells pretreated with PBS (ctrl) or 10 µM 187-1 (+187-1). Values relative to PBS pretreated WT MEFs and Hap1 cells respectively. Values are the means of three independent replicates (each replicate is comprised of 6 fields of view, n=18. Each field of view is approximately 50-100 cells, see methods). Error bars indicate standard deviation. NS – no significant difference, ** - P <0.01 (ANOVA followed by post hoc Dunnett's comparison) **(D)** Immunoblot confirming ectopic expression in WT MEFs of N-WASP, N-WASP^{L299P} and PH-VCA, with tubulin shown as a loading control. **(E)** Immunoblot confirming ectopic expression in WT MEFs of Cdc42^{QL} and Rac1^{QL}, with tubulin shown as a loading control.

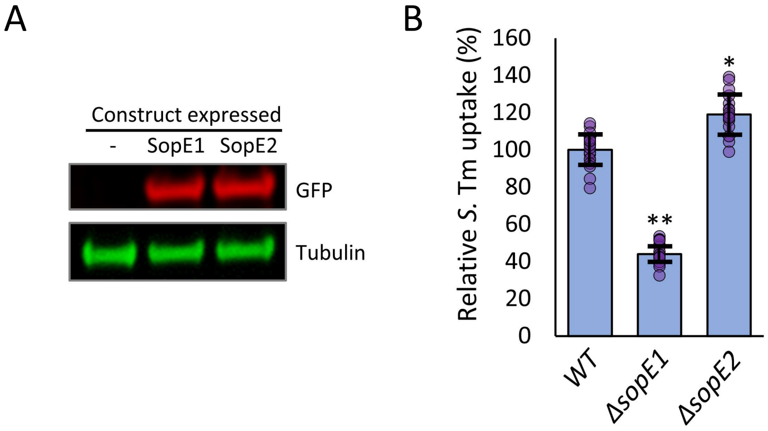

Figure S2: N-WASP dependent inhibition of *Salmonella* uptake is driven by SopE2. Related to Figure 2. (A) Immunoblot confirming ectopic expression in WT MEFs of SopE1 and SopE2, with tubulin shown as a loading control. **(B)** Uptake of WT, Δ*sopE1* and Δ*sopE2 S. Tm* (30 minutes) into WT Hap1 cells. Values relative to WT *S.* Tm uptake. Values are the means of three independent replicates (each replicate is comprised of 6 fields of view, n=18. Each field of view is approximately 50-100 cells, see methods). Error bars indicate standard deviation. NS – no significant difference, ** - P <0.01, * - P<0.05 (ANOVA followed by post hoc Dunnett's comparison).

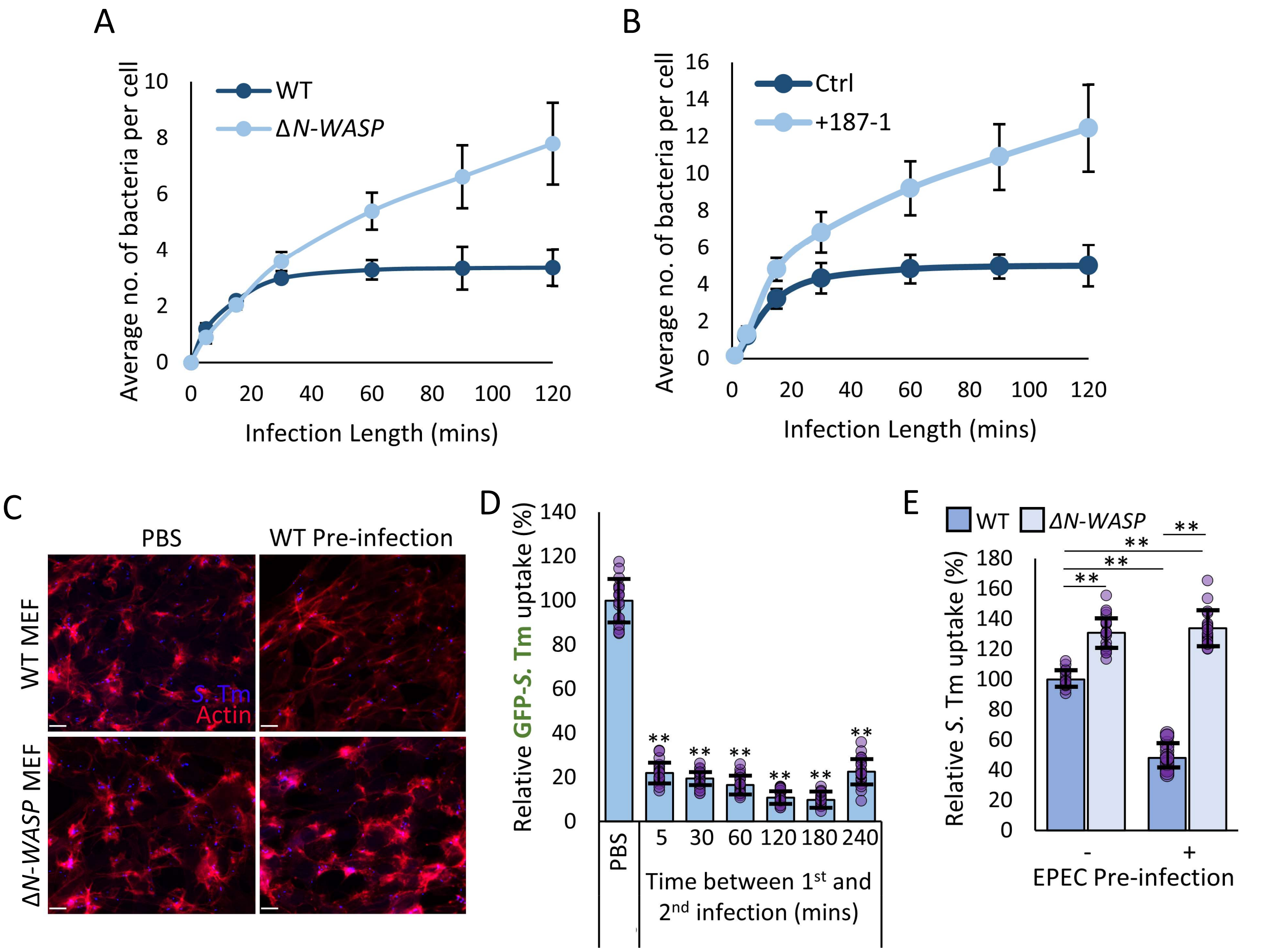

Figure S3: SopE2 and N-WASP inhibit *Salmonella* uptake at later timepoints of invasion. **Related to Figure 3. (A)** Average number of WT *S.* Tm taken up into WT and ΔN-WASP Hap1 cells MEFs after indicated infection length. **(B)** Average number of WT *S.* Tm taken up into WT MEFs pretreated with PBS (ctrl) or 187-1 (+187-1) MEFs after indicated infection length. **(C)** Fluorescence microscopy images of WT and ΔN-WASP MEFs either pretreated with PBS or pre-infected for 1 hour with unstained WT *S.* Tm, and then infected with stained WT S. Tm (blue) for 5 minutes. Actin (and ruffles formed by *S.* Tm) visualised using Texas-Red Phalloidin (red) **(D)** Uptake of WT GFP-expressing *S. Tm* into WT MEF cells that had been pre-infected with WT *S.* Tm (60 minutes). After the first infection cells were incubated in media containing Gentamycin for the indicated time before performing the 2nd infection with GFP-expressing *S. Tm*. All values relative to uptake into PBS pretreated control cells. **(E)** Uptake of WT S. *Tm* into WT and ΔN-WASP MEF control cells (-) or cells that had been pre-infected for 90 minutes with WT EPEC (+). Values relative to uptake into control WT MEFs.

All *S.* Tm uptake values are the means of three independent replicates (each replicate is comprised of 6 fields of view, n=18. Each field of view is approximately 50-100 cells, see methods). Error bars indicate standard deviation. NS – no significant difference, ** - P <0.01 (ANOVA followed by post hoc Dunnett's comparison)

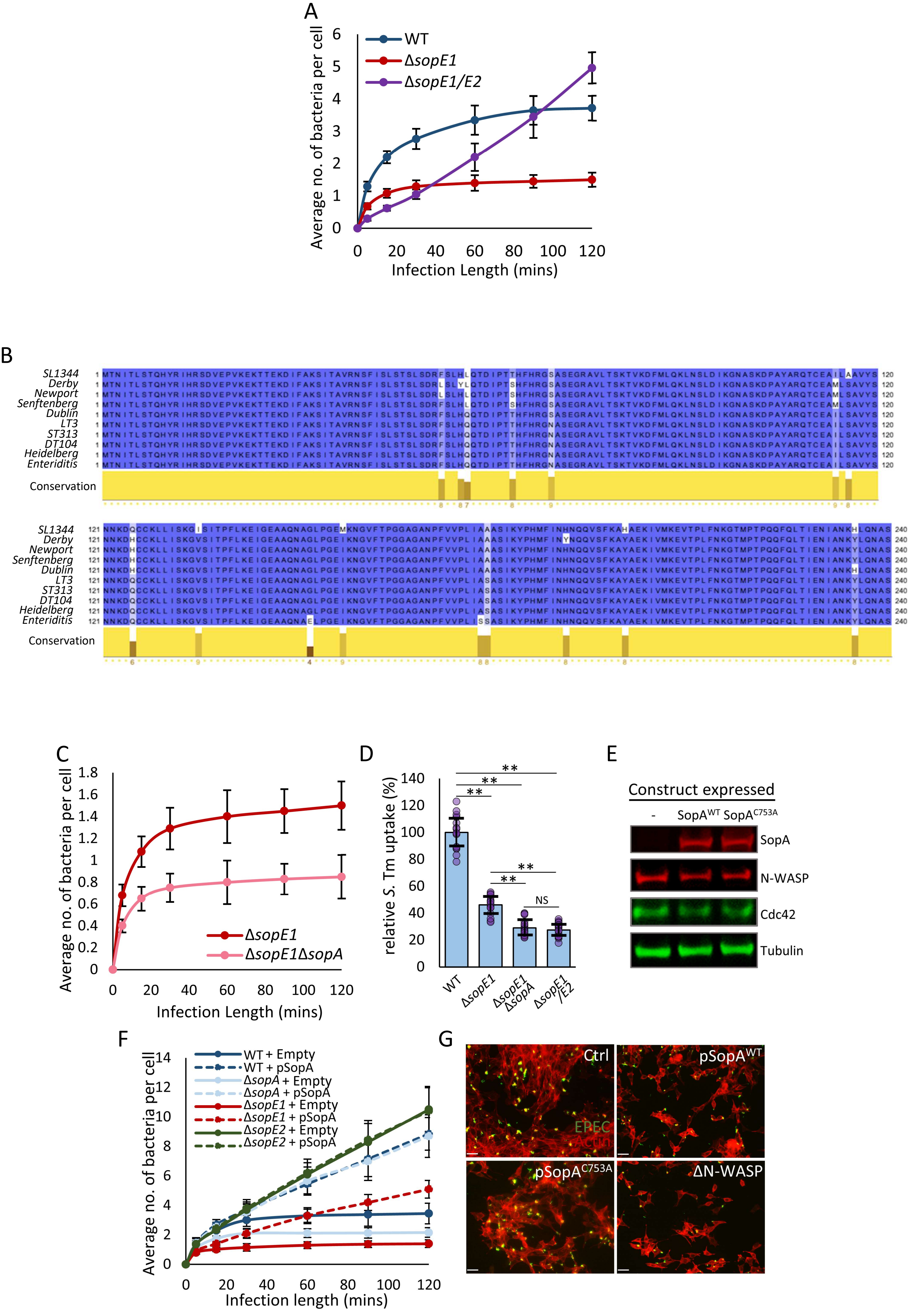
A
Average no. of bacteria per cell
Infection Length (mins)
WT
ΔsopE1
ΔsopE1/E2
B
SL1344
Derby
Newport
Senftenberg
Dublin
LT3
ST313
DT104
Heidelberg
Enteriditis
Conservation
C
Average no. of bacteria per cell
Infection Length (mins)
ΔsopE1
ΔsopE1ΔsopA
D
relative S. Tm uptake (%)
**
NS
WT
ΔsopE1
ΔsopE1 ΔsopA
ΔsopE1 /E2
E
Construct expressed
-
SopA^WT
SopA^C753A
SopA
N-WASP
Cdc42
Tubulin
F
Average no. of bacteria per cell
Infection length (mins)
WT + Empty
WT + pSopA
ΔsopA + Empty
ΔsopA + pSopA
ΔsopE1 + Empty
ΔsopE1 + pSopA
ΔsopE2 + Empty
ΔsopE2 + pSopA
G
Ctrl
pSopA^WT
pSopA^C753A
ΔN-WASP
EPEC
Actin

Figure S4: SopA controls how SopE2 affects *Salmonella* uptake. **Related to Figure 4 (A)** Average number of WT, Δ*sopE1* and Δ*sopE1/E2 S.* Tm taken up into WT MEFs after indicated infection length. **(B)** Sequence alignment of SopE2 proteins from multiple *Salmonella* strains. Alignment was generated using Custal Omega using the following sequences: SL1344, *S. enterica* serovar Typhimurium strain SL1344 (Uniprot A0A718RQV9); Derby, *S. enterica* serovar Derby (Uniprot A0A739PYC9); Newport, *S. enterica* serovar Newport (Uniprot A0A737BC43); Senftenberg, *S. enterica* serovar Senftenberg (Uniprot A0A3V2HX07): Dublin, *S. enterica* serovar Dublin (Uniport A0A732GUY5); LT2, *S. enterica* serovar Typhimurium strain LT2 (Uniprot Q7CQD4); ST313, *S. enterica* serovar Typhimurium sequence type 313 strain b3589 (Genbank THC71077.1); DT104, *S. enterica* serovar Typhimurium strain DT104 (Genbank CCW74528.1); Heidelberg, *S. enterica* serovar Heidelberg (Genbank QQH45576.1); Enteritidis, *S. enterica* serovar Enteritidis (Uniprot A0A6Y0U861). Conservation (0 least conserved, 9 most conserved) was assessed in Jalview and displayed beneath the sequence alignment. **(C)** Average number of Δ*sopE1* and Δ*sopE1*Δ*sopA S.* Tm taken up into WT MEFs after indicated infection length. Data are from Fig 4C. **(D)** Uptake of WT, Δ*sopE1* , Δ*sopE1*Δ*sopA* and Δ*sopE1/E2 S.* Tm after 15-minute infection of WT MEFs. Values relative to WT *S. Tm* uptake. **(E)** Immunoblot confirming ectopic expression in WT MEFs of $SopA^{WT}$ and $SopA^{C753A}$. Also shown are relative levels of N-WASP, Cdc42 and loading control tubulin. **(F)** Average number of WT, Δ*sopA*, Δ*sopE1* and Δ*sopE2 S.* Tm taken up into WT MEFs ectopically expressing and empty control vector (+Empty) or SopA (+pSopA) after indicated infection length. **(G)** Representative immunofluorescence images of EPEC attachment quantified in Fig 4G. WT (Ctrl), Δ*N-WASP* MEFs (Δ*N-WASP*) or WT MEFs ectopically expressing either SopA ($pSopA^{WT}$) or $SopA^{C753A}$ ($pSopA^{C753A}$) were infected with WT EPEC for 90 minutes, and cells then washed in PBS and 200 mM glycine pH 2 to remove poorly adherent bacteria. Cells were stained with anti-intimin antibody to visualise EPEC (green) and Texas-Red Phalloidin to visualise actin (red). Scale bar 20 µm.

All *S.* Tm uptake values are the means of three independent replicates (each replicate is comprised of 6 fields of view, n=18. Each field of view is approximately 50-100 cells, see methods). All error bars indicate standard deviation. NS – no significant difference, ** - P <0.01. (ANOVA followed by post hoc Dunnett's comparison).

Table S1: Oligonucleotides used in this paper. Related to STAR methods

Oligonucleotide	Source
**P1**<br>TTGAAAGCAAGAAATATAAACAAAGTGTAGCTAT GCATAGTTATCTAAAAGGAGAACTACCGTGATTCCGGGGATCCGTCGACC<br>*sopE2 deletion Forward Primer*	This Paper
**P2**<br>ATTAATTCATATGGTTAATAGCAGTATTGTATTTACTACCATCAGGAGGCATTCTGAAGATATGTAGGCTGGAGCTGC TTCG<br>*sopE2 deletion Reverse Primer*	This Paper
**P3**<br>GGAAAATAAAATTTATAAATATCAATGAG<br>sopE2 Screening Primer Forward	This Paper
**P4**<br>GATTTTTATATTAAAATAAAGATGACGACG<br>sopE2 Screening Primer Reverse	This Paper
**P5**<br>ACTTTTAAGGCGTTAAAAATCCAGACCGTTTTTCCATAATGATGTTGATAAGGAATTCTAATGAGGGTTTTCCCAGTCACGAC<br>sopA deletion forward primer	This Paper
**P6**<br>GGACACAACGCTGTGTCCCTTAATTCCATGCGGG TTGAGGCTGGACTACGCCCAGGCCAGTGGTGCTT CCGGCTCGTATGTTG<br>sopA deletion reverse primer	This Paper
**P7**<br>AGAAAGCCGGAGAAACCCTTACG<br>sopA Screening Primer Forward	This Paper
**P8**<br>CAAGATACTGTTCATAAAGCGCTGC<br>sopA Screening Primer Reverse	This Paper
**P9**<br>TAAAAGCATTCCTGCTATCTATATATAAATGAATT ATGTACATATAAAAGGATCATTACCGTGATTCCG GGGATCCGTCGACC<br>sopE1 deletion forward Primer	This Paper
**P10**<br>TATTAATCAGGAAGAGGCTCCGCATATTTTTTGG TTTTTCAGTGTTCAGGGAGTGTTTTGTATTGTAG GCTGGAGCTGCTTCG<br>sopE1 deletion Reverse Primer	This Paper
**P11**<br>CTATTTGTAGCATTAATTGAATCAGC<br>sopE1 Screening Primer Forward	This Paper
**P12**<br>CTATATGTTATATAGCAGCAATGGC<br>sopE1 Screening Primer Reverse	This Paper
**SopA C753A F**<br>CCTCCATGGCGCCTTTACCGCGACCAGCGTTGT GGCGGATAG<br>SDM Forward primer to generate SopA^{C753A}	This Paper
**SopA C753A R**<br>CTATCCGCCACAACGCTGGTCGCGGTAAAGGC GCCATGGAGG<br>SDM Reverse primer to generate SopA^{C753A}	This Paper
**N-WASP L299P F**<br>AATAATTTGGATCCAGAACCGAAGAATCTTTTTGATATG<br>SDM Forward primer to generate N-WASP^{L299P}	This Paper
**N-WASP L299P R**<br>CATATCAAAAAGATTCTTCGGTTCTGGATCCAAATTATT<br>SDM Reverse primer to generate N-WASP^{L299P}	This Paper
**N-WASP GW F**<br>GGGGACAAGTTTGTACAAAAAAGCAGGCTCCAGCTCG<br>GGCCAGCAGC<br>Gateway Forward Primer to generate pEmerald-N-WASP	This Paper
**N-WASP GW R**<br>GGGACCACTTTGTACAAGAAAGCTGGGTCTCAGTCTTC CCACTCATCATC<br>Gateway Reverse Primer to generate pEmerald-N-WASP	This Paper

**Cdc42 GW F**<br>GGGGACAAGTTTGTACAAAAAAGCAGGCTCCCAGACA ATTAAGTGTGTTG<br>Gateway Forward Primer to generate pEmerald-Cdc42 and pEmerald Cdc42^{QL}	**This Paper**
**Cdc42 GW R**<br>GGGACCACTTTGTACAAGAAAGCTGGGTCTCATAGCAG CACACACCTGCGGCTC<br>Gateway Reverse Primer to generate pEmerald-Cdc42 and pEmerald-Cdc42^{QL}	This Paper
**Rac1 GW F**<br>GGGGACAAGTTTGTACAAAAAAGCAGGCTCCCAGGC CATCAAGTGTG<br>Gateway Forward Primer to generate pEmerald-Rac1^{QL}	This Paper
**Rac1 GW R**<br>GGGACCACTTTGTACAAGAAAGCTGGGTCTTACAACA GCAGGCATTTTCTCTTC<br>Gateway Reverse Primer to generate pEmerald Rac1^{QL}	This Paper
**SopE1 78 GW F**<br>GGGGACAAGTTTGTACAAAAAAGCAGGCTCCTTGACA AATAAAGTCGTTA<br>Gateway Forward Primer to generate pEmerald-SopE1$^{78-241}$	This Paper
**SopE1 GW R**<br>GGGACCACTTTGTACAAGAAAGCTGGGTCTCAGGGAG TGTTTTGTATATA<br>Gateway Reverse Primer to generate pEmerald-SopE1$^{78-241}$	This Paper
**SopE2 70 GW F**<br>GGGGACAAGTTTGTACAAAAAAGCAGGCTCCATGGGG AACGCTTCTGAGG<br>Gateway Forward Primer to generate pEmerald-SopE2$^{70-240}$	This Paper
**SopE2 GW R**<br>GGGACCACTTTGTACAAGAAAGCTGGGTCTCAGGAGGC ATTCTGAAGATA<br>Gateway Reverse Primer to generate pEmerald-SopE2$^{70-240}$	This Paper
**SopA 60 GW F**<br>GGGGACAAGTTTGTACAAAAAAGCAGGCTCCTTCGCGT TACCACAAAAAA<br>Gateway Forward Primer to generate pHA-SopA$^{60-782}$	This Paper
**SopA GW R**<br>GGGACCACTTTGTACAAGAAAGCTGGGTCCTACGCCCA GGCCAGTGGCAG<br>Gateway Reverse Primer to generate pHA-SopA$^{60-782}$	This Paper
